# An Omnidirectional Vision Sensor Based on a Spherical Mirror Catadioptric System

**DOI:** 10.3390/s18020408

**Published:** 2018-01-31

**Authors:** Sandro Barone, Marina Carulli, Paolo Neri, Alessandro Paoli, Armando Viviano Razionale

**Affiliations:** 1 Department of Civil and Industrial Engineering, University of Pisa, Largo Lucio Lazzarino 1, 56122 Pisa, Italy; s.barone@ing.unipi.it (S.B.); a.paoli@ing.unipi.it (A.P.); a.razionale@ing.unipi.it (A.V.R.); 2 Dipartimento di Meccanica, Politecnico di Milano, Via Giuseppe La Masa, 1, 20156 Milano, Italy; marina.carulli@polimi.it

**Keywords:** catadioptric sensor, forward projection model, backward projection model, spherical mirror, computer vision

## Abstract

The combination of mirrors and lenses, which defines a catadioptric sensor, is widely used in the computer vision field. The definition of a catadioptric sensors is based on three main features: hardware setup, projection modelling and calibration process. In this paper, a complete description of these aspects is given for an omnidirectional sensor based on a spherical mirror. The projection model of a catadioptric system can be described by the forward projection task (FP, from 3D scene point to 2D pixel coordinates) and backward projection task (BP, from 2D coordinates to 3D direction of the incident light). The forward projection of non-central catadioptric vision systems, typically obtained by using curved mirrors, is usually modelled by using a central approximation and/or by adopting iterative approaches. In this paper, an analytical closed-form solution to compute both forward and backward projection for a non-central catadioptric system with a spherical mirror is presented. In particular, the forward projection is reduced to a 4th order polynomial by determining the reflection point on the mirror surface through the intersection between a sphere and an ellipse. A matrix format of the implemented models, suitable for fast point clouds handling, is also described. A robust calibration procedure is also proposed and applied to calibrate a catadioptric sensor by determining the mirror radius and center with respect to the camera.

## 1. Introduction

Catadioptric sensors are defined by integrating mirrors and cameras. In particular, the use of external curved mirrors to reflect the scene into the camera lens provides an increased field of view with respect to traditional camera systems, thus allowing the definition of omnidirectional devices [1]. The viewed scene undergoes a transformation due to the reflection in the mirror. A mapping function (projection model) enables the re-projection of 3D points on the scene to the corresponding 2D camera pixels. If the scene is observed from a single point in the space, the sensor has a unique center of projection (central projection) and the mapping function is easy to be modelled and used. Nevertheless, the use of curved mirrors leads to central catadioptric sensors (effective single viewpoint) only under severe constraints on the camera placement with respect to the mirror [2]. When light rays do not intersect in a single point, the imaging system does not maintain a single viewpoint and a locus of viewpoints (caustic) in the three-dimensional space is created [3]. Non-central catadioptric sensors increase the flexibility in the positioning of the imaging devices but introduce difficulties in the analytical modelling of the mapping function. 

A practical solution to create a non-central catadioptric sensor consists in the use of a spherical mirror, which is relatively easy to manufacture. The projection model can be described by the forward projection (FP) task, which is represented by the computation of the 2D projection on the image plane of a 3D scene point. The inverse problem (backward projection, BP) consists in the determination of the 3D scene point, or direction of the incident light, starting from the 2D image pixel coordinates. The phenomenon of the light reflection on a specular surface is well known and usually approached by exploiting the Reflection Law: at the reflection point on a perfect specular surface, the incident ray, the reflected ray and the surface normal vector are disposed on the same plane and the surface normal vector bisects the angle between incident and reflected rays [4]. When the direction of incident or reflected ray is known, the reflection point can be computed by determining the intersection with the mirror surface and resolving the other direction by using the Reflection Law. The BP problem can be solved in a closed-form since the direction of projection, starting from the 2D camera point, can be determined with a classical pinhole model. On the other hand, the FP task is more challenging since neither incident and reflected directions are known. Many models are available in technical literature [2] exploiting different approaches. However, within the huge variety of existent solutions, many are essentially designed for single viewpoint configurations and their application to non-central or slightly non-SVP configurations (as spherical mirrors) introduces approximations [5,6].

The present paper proposes a complete description of a complex problem, which is of great interest in the computer vision field. The main contribution consists in outlining a methodological approach for catadioptric systems based on spherical mirrors, from problem modelling to hardware setup through software implementation. The developed tools exploit an exact analytical closed-form solution for both BP and FP tasks in the case of a non-central catadioptric sensor composed of a perspective camera and a spherical mirror. No restrictions are introduced on the mirror solid angle nor on the relative placement between the camera and the mirror. Moreover, the described projection model has been implemented as an open source code.

## 2. Background

It is assumed that closed-form or analytical solutions relating 3D world point coordinates to their corresponding image coordinates do not exist for mirrors with a general shape [7]. Iterative methods (i.e., bundle adjustment) are usually adopted by minimizing cost functions and assuming the specular surface described by a quadratic expression. Standard 2D re-projection errors [8] or angular errors between incident and reflected rays [9] can be used as cost functions. An analytical FP model for the projection of a 3D point reflected by a generic quadric mirror, with no restrictions on the camera placement, is presented in [10]. The reflection point can be obtained by solving an 8th degree polynomial in a single parameter, whose coefficient expressions are computed in a quasi-closed-form. An analytical solution to compute the FP for axial configurations, where the mirror is obtained by revolving a conic section around the axis of symmetry and the camera center of projection is placed on the mirror axis, is presented in [11]. This approach demonstrates that for a given 3D point, the mirror reflection point can be obtained by solving a 6th degree form. An analytical solution for mirror surfaces described by a quadratic expression is also proposed in [7]. The authors demonstrated that the reflection point can be iteratively searched on a single parameter curve in the space given by the intersection of two quadratic equations. 

The special case of determining the mirror reflection point for a spherical mirror is popularly known as the Alhazen’s problem [10]. Some approaches to compute direct reflections on spherical mirrors with high accuracy and high performances have been proposed. For example, an approximated non-closed-form solution, obtained by relaxing the reflection law and minimizing the introduced errors by means of bundle adjustment, is presented in [12]. A closed-form solution to solve the mapping process between world points and their equivalent image points is proposed in [13]. However, two assumptions are made: the center of the spherical mirror must belong to the optical axis and the mirror must be at least a hemisphere visible by the camera. An analytical solution for the FP problem, reduced to a 4th degree equation and without any restriction on the camera placement, is presented in [10,11]. The system to be solved is determined by imposing that the reflection point must belong to the mirror surface and that the reflected ray (obtained by using the reflection law) must pass through the camera center. Finally, a novel approach, which exploits an iterative search of the reflection point (i.e., non-closed-form solution) is proposed in [14] and the relative performances are compared to those obtained by the FP model presented in [10].

**Figure 1:**
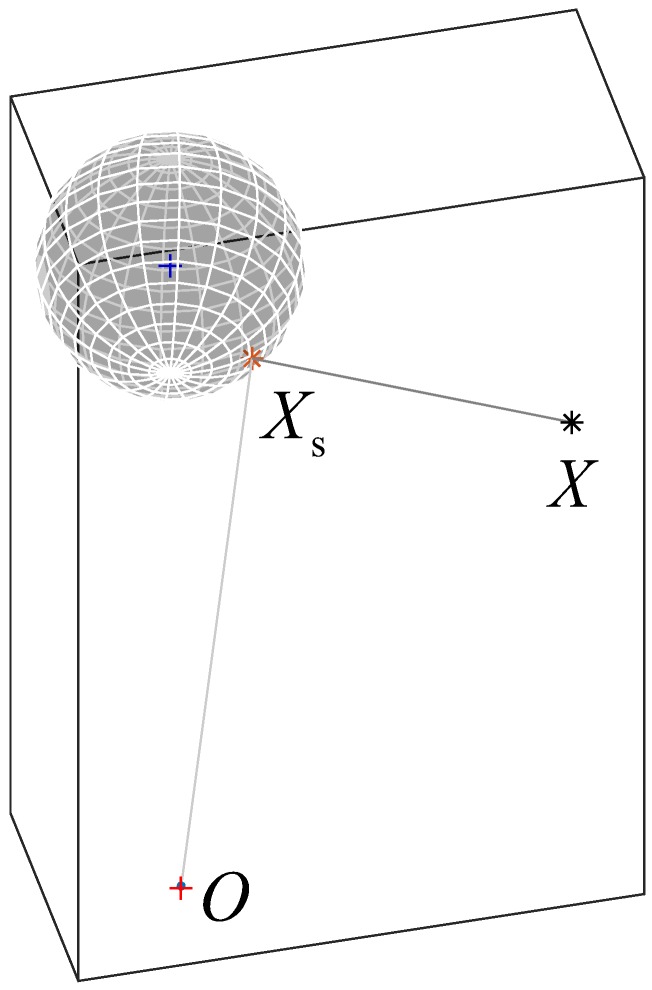
Reflection scheme for a single 3D point.

The present paper presents an analytical closed-form solution for a catadioptric sensor composed of a perspective camera and a spherical mirror as shown in Figure 1. The red cross represents the camera central point, the red star represents the reflection point while the black star represents the projected 3D point. 

No restrictions are introduced on the relative placement between the optical axis and the center of the spherical mirror and the mirror can be a spherical cap smaller than a hemisphere. In particular, a simplified formalization for the FP model is presented and discussed. The proposed approach is based on the analytical computation of the reflection point, similarly to [10,11]. However, the approach proposed in [10,11] exploits the Reflection Law and imposes that the reflected ray must pass through the camera center. In the present work, the intersection between the spherical mirror and an ellipse is rather found, thus allowing an easier parametrization and simpler equations. The adopted parametrization allows to reduce the problem to a 4th order polynomial, thus representing the same physical solution. A straightforward evaluation procedure allows to automatically select the proper solution among the four available. 

The proposed solution scheme was also implemented in a matrix format, for fast point-clouds handling. Many practical applications in the computer vision field may benefit, indeed, from the use of an accurate and fast projection model for non-SVP catadioptric systems. For example, the FP model can be used for rendering reflections, camera calibration tasks and pose estimation, while the backward projection model can be used for 3D reconstruction processes. In this work, the proposed projection model has been used to calibrate a catadioptric sensor by determining the spherical mirror radius and center with respect to the camera. At this purpose, the Jacobian matrices of both FP and BP functions were computed and their role in the optimization procedures for the calibration process discussed in the paper. The developed projection functions have been also implemented as Matlab scripts (“*cam2world.m*” and “*world2cam.m*”), which are available as supplementary material at [15].

## 3. Proposed Projection Model

Given a pinhole camera, a spherical mirror and a 3D point, it is possible to find an analytical closed-form solution for both backward and forward projection tasks.

### 3.1. Backward Projection Model

**Figure 2:**
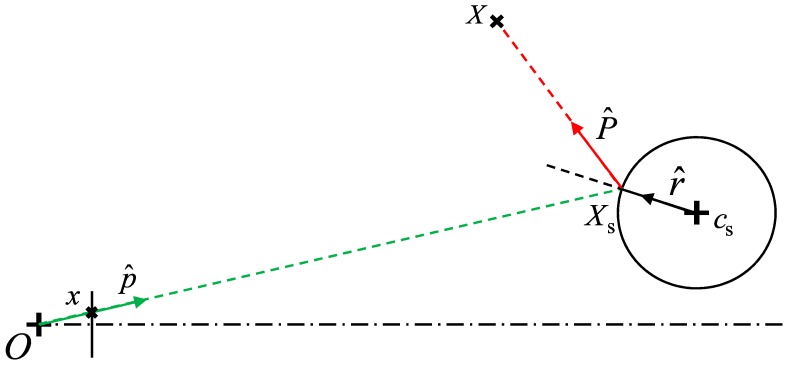
Backward projection geometrical scheme.

The simpler backward projection solution is presented first, using the scheme in Figure 2 as a reference, which may be interpreted as a section view of the scheme in Figure 1. The origin *O* is placed on the camera central point and the mirror center, *c*_s_, is a 3D point in the space without any restriction (it is not required to belong to the camera optical axis, which is represented with a dash-dot line in Figure 2). In the following, the hat symbol “$\hat{.}$” denotes a unit vector. The point *x* represents, in pixels, the 2D location on the camera image plane of the 3D projected point *X*. The projection direction $\hat{p}$ can be easily determined by the classical pinhole model, taking into account also terms for radial and tangential distortions [2].

It is then possible to find the 3D reflection point, *X*_s_, by searching for the intersection between the line passing through the origin with the direction $\hat{p}$ and the sphere (having center *c*_s_ and radius *r*). The point *X*_s_ can be expressed as $\hat{p}d$, being *d* the distance between *X*_s_ and *O*. Since *X*_s_ lies on the sphere surface, it is possible to state that |*X*_s_ − *c*_s_|^2^ = *r*^2^, which can be rewritten as:

(1) $$d^{2}+|c_{s}{|}^{2}-2d({\hat{p}}^{T}\cdot c_{s})-r^{2}=0$$

where “·” represents the scalar product between the 3D vectors. Equation (1) is a second-degree scalar equation having only one unknown, *d*. Two solutions can then be found:

(2) $$d=({\hat{p}}^{T}\cdot c_{s})\pm \sqrt{{\left({\hat{p}}^{T}\cdot c_{s}\right)}^{2}-(|c_{s}{|}^{2}-r^{2})}$$

The “−” solution must be selected since the physical correct solution is determined by the closest point with respect to the origin. The direction of the projection ray $\hat{P}$ can be directly determined by considering that the radius passing by *X*_s_ must be the bisector of the angle between $\hat{p}$ and $\hat{P}$ (Reflection Law). Thus, the sum $\hat{P}+(-\hat{p})$ gives a vector aligned with the radius $\hat{r}$ and having a modulus equal to the double of the projection of $-\hat{p}$, i.e., $-\hat{p}\hat{r}$. Hence, it holds:

(3) $$\hat{P}+(-\hat{p})=2(-{\hat{p}}^{T}\cdot \hat{r})\hat{r}$$

Solving this equation for $\hat{P}$, the backward projection problem for the point *x* is determined in a closed-form, since the 3D point *X* lies on the line passing by *X*_s_ and having the direction $\hat{P}$.

### 3.2. Forward Projection Model

**Figure 3:**
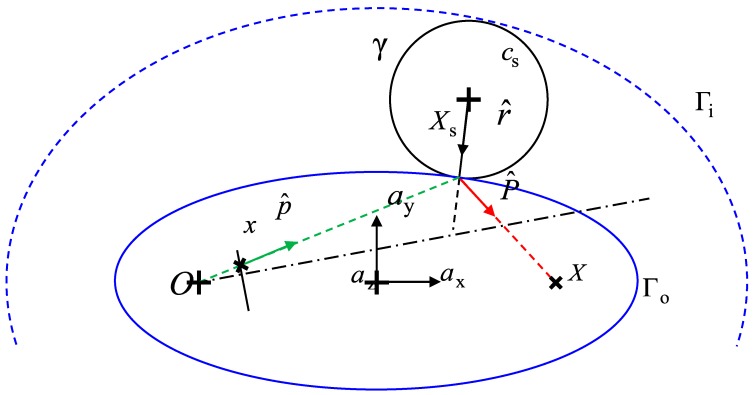
Forward projection geometrical scheme.

Forward projection is more complex since neither direction $\hat{P}$ nor $\hat{p}$ are known. For any 3D point *X*, the solution of this problem lies on the plane passing by *X*, *O* and *c*_s_. The normal to this plane can be easily determined by computing the cross product between $\vec{OX}$ and $\vec{c_{S}X}$. It is possible to demonstrate that the reflection point *X*_s_ is the intersection between the circle *γ* centered in *c*_s_ (i.e., the sphere section with the solution plane) and an ellipse (*Г*_i_ for concave mirrors or *Г*_o_ for convex mirrors, which is the case of the present paper) having *X* and *O* as foci and tangent to the circle [16], as shown in Figure 3. A reference system is defined with the origin placed at the midpoint between the two foci, the *z*-axis (*a*_z_) normal to the solution plane and the *x*-axis (*a*_x_) aligned with the $\vec{OX}$ direction (the *y*-axis *a*_y_, consequently, is determined by the cross product of the other two directions). These three unit vectors define a rotation, which converts the 3D coordinates of any point between the global reference frame (which is the camera reference frame) and the solution reference frame.

All the coordinates can be scaled by the factor $s_{f}=\left|\vec{OX}\right|/2$ for convenience. Thus, the origin *O* and the point *X* can be expressed as [−1, 0, 0] and [1, 0, 0] respectively (note that the reference frame was centered at the midpoint between *O* and *X*) and *c*_s_/*s*_f_ = [*a*, *b*, 0]^T^ (*z*-coordinate is equal to 0 since the center belongs to the solution plane). It is then possible to show that the intersection between the circle *γ* and the tangent ellipse *Г*_o_ lies on the cubic (see Appendix A):

(4) (ay−bx)(x(x−a)+y(y−b))−(x−a)(y−b)=0

where *x* and *y* represent coordinates scaled by the factor *s*_f_. A further reference frame, centered in *c*_s_, is also considered in order to express *x*/*y*-coordinates as *w* = *x* − *a*, *q* = *y* − *b*. The reflection point *X*_s_ can then be found by looking for the intersection between the cubic curve expressed by Equation (4) (substituting *w* and *q*) and the circle *γ*:

(5) $$\begin{array}{l} (aq-bw)(aw+bq+r^{2})-wq=0,\\ w^{2}+q^{2}=r^{2}. \end{array}$$

The second equation of the system can also be expressed in polar coordinates, so that *w* = *r* cos(*ϑ*) and *w* = *q* sin(*ϑ*), being *r* the radius of the mirror surface. If these expressions are substituted in the first of Equations (5), and the well-known parametrization of sine and cosine is adopted (cos(*ϑ*) = (1 − *t*^2^)/(1 + *t*^2^), sin(*ϑ*) = 2*t*/(1 + *t*^2^)), the following expression is obtained:

(6) $$(br-ab)t^{4}+2(ar+b^{2}-a^{2}+1)t^{3}+6(ab)t^{2}+2(ar-b^{2}+a^{2}-1)t+(-br-ab)=0$$

This expression is a 4th order polynomial in the variable *t*, which obviously can be solved in a closed-form [17] giving four different solutions. These solutions represent all the possible intersections between the circle and the generic quadric *x*^2^/(1 + *λ*) + *y*^2^/*λ* = 1. The variable *λ* = (*x* − *a*)*y*/(*ay* − *bx*) can be used to parametrize the generic quadric tangent to the circle, such that *λ >* 0 corresponds to an ellipse and −1 < *λ* < 0 corresponds to a hyperbola. Thus, the computation of the parameter *λ* allows to firstly select the two solutions with *λ* > 0, in order to exclude the hyperbola from the results. The two remaining solutions represent two ellipses tangent to the given circle: the inner tangent *Г*_i_ (blue dashed line in Figure 2) and the outer tangent *Г*_o_ (blue solid line in Figure 2). The physically correct solution is the one described by the outer tangent ellipse *Г*_o_, which is characterized by the smaller principal axis, and can be found by looking for the minimum of the parameter *λ* (smallest positive value). Once the proper solution is identified, *X*_s_ can be found by substituting back the sine/cosine parametrization and multiplying them by the sphere radius. It is then possible to transform the coordinates in the global reference system and re-project 3D points onto the image plane by using the classical pinhole model. Figure 1 reports the projection scheme for a real case with the relative placement between spherical mirror and camera described in Section 6.

## 4. Software Implementation

The previous paragraph describes how forward and backward projection can be analytically solved in a closed-form for a single point. If large data are considered, i.e., a large number of points, a *for*-loop can be carried out by sequentially perform the computation on the various points singularly. Anyway, matrix formulation can speed up several operations, depending on the implementation and on the number of points. The present section describes the main steps required to implement the described models in order to process arrays of points. The performances of the *for*-loop were finally compared to the performances of the proposed matrix implementation for both BP and FP tasks with an increasing number of points to be projected.

### 4.1. Matrix Format

In many practical applications (i.e., camera calibration or 3D reconstruction) a great number of points must be handled by the projection functions. For this reason, the proposed formulation could greatly benefit from the introduction of a matrix format in order to speed up calculation tasks. In the following, the adopted notations and conventions are referred to the Matlab software (version: Matlab 2016b), which has been used to develop scripts for both forward and backward projection problems [15]. In the present work, the coordinates of each point (2D or 3D) are expressed by a column vector, so that they can be arranged in a matrix having a number of rows corresponding to the point dimension (i.e., 2 for “*cam2world.m*” function, 3 for “*world2cam.m*” function) and a number of columns corresponding to the number of points.

The backward projection problem only requires the solution of a second-order equation, whose coefficients, for a single point, can be defined as:

(7) $$\begin{array}{l} a_{1}=1,\\ a_{2}=-2{\hat{p}}^{T}\cdot c_{s},\\ a_{3}=\;|c_{s}{|}^{2}-r^{2}. \end{array}$$

In the case of multiple (*n*) projected points, both *a*_1_ and *a*_3_ have the same values for all the points. On the other hand, *a*_2_ has a different value for each projected point, so that a column vector can be obtained by computing the product $-2{\hat{p}}^{T}\cdot c_{s}$, where $\hat{p}$ is now a 3 × *n* matrix having the vectors ${\hat{p}}_{i}$ as columns. Equation (2) can be directly solved obtaining the vector *d*, which contains the distances from the origin of the reflection points corresponding to each projected point. It is then possible to build a 3 × *n* matrix composed by the repetition of *d*^T^: the element-by-element multiplication between this matrix and $\hat{p}$ provides the 3D coordinates of the reflection points, *X*_s,i_, which are again grouped in the 3 × *n* matrix **X_s_**. Finally, the directions of the reflected rays can be obtained by solving Equation (3), where $\hat{r}$ is a 3 × *n* matrix obtained by subtracting *c*_s_ from each column of the matrix **X_s_**, and then normalizing each column.

The FP problem has a more complex formulation, since a proper coordinate system must be defined for each point. The *z*-axes (*a*_z_) are represented by a 3 × *n* matrix **a_z_** which may be calculated by the cross product:

(8) $$a_{z}=\left[X_{1},X_{2},\ldots ,X_{n}\right]\times \left[c_{s},c_{s},\ldots ,c_{s}\right]=X\times \left[c_{s},c_{s},\ldots ,c_{s}\right]$$

where *X_i_* (*i* = 1,…,*n*) are the vectors representing each projected 3D point and *c*_s_ is the vector representing the sphere center (the column-wise cross product is carried out). The *x*-axes are collected in a matrix **a_x_**, which can be easily determined by the 3 × *n* matrix **X** and, thus, the *y*-axes are obtained through the column-wise cross product between matrices: **a_y_** = **a_z_** × **a_x_**. Finally, the so-defined three matrices **a_x_**, **a_y_**, **a_z_** are column-wise normalized in order to determine the matrices ${\hat{a}}_{x}$, ${\hat{a}}_{y}$, ${\hat{a}}_{z}$ (each column of these matrices is a unit vector). These 3 × *n* matrices contain the normalized directions of the reference systems corresponding to the solution plane for each projected point. Thus, the *i*-th rotation matrix can be determined as $R_{i}=\left[\begin{array}{ccc} {\hat{a}}_{x,i} & {\hat{a}}_{y,i} & {\hat{a}}_{z,i} \end{array}\right]$ (where ${\hat{a}}_{k,i}$ is the *i*-th column of the matrix ${\hat{a}}_{k}$, for *k* = *x*, *y*, *z*). These rotation matrices are arranged in a block diagonal matrix, **R_g_**, in order to avoid any *for*-loop in the code. Finally, since the origins of the solution reference frames are moved at the midpoint between *O* and the 3D point to be projected, a translation matrix can be defined as **T** = [*T*_1_ *T*_2_ ··· *T_n_*] = **X**/2.

According to Equation (6), the coefficients of the 4th order polynomial form to be solved only depend on the coordinates of the sphere’s center in the local coordinate system (*c*_p_). For this reason, they assume different values for each projected point, which can be obtained by computing:

(9) $$\begin{array}{l} R_{g}=\left[\begin{array}{cccc} R_{1} & 0 & \cdots & 0\\ 0 & R_{2} & \cdots & 0\\ \vdots & \vdots & \ddots & \vdots \\ 0 & 0 & \cdots & R_{n} \end{array}\right],\\ c_{p}=R_{g}\left[\begin{array}{c} c_{s}-T_{1}\\ c_{s}-T_{2}\\ \vdots \\ c_{s}-T_{n} \end{array}\right]. \end{array}$$

It is worth noting that **R_g_** has large dimensions (i.e., 3*n* × 3*n*) while many operations could be accomplished by using the 3*n* × 3 matrix obtained by arranging **R*_i_*** in a column block vector. However, this more complete notation has been used for the rotation computation since it turns useful for the Jacobian computation (see below). Moreover, this notation allows to easily define the inverse rotation matrix by transposing the block diagonal matrix **R_g_**. The vector *c*_p_ has 3*n* elements and can then be rearranged in the 3 × *n* matrix ***c_p_***. Anyway, the third row of the matrix is composed of 0 terms since the sphere’s center lies on the solution plane, and thus can be neglected. All the coefficients needed for the 4th order polynomial solution are obtained by solving Equation 6 in an element-by-element fashion, only requiring the first two rows of **c_p_**. The result of this calculation is determined by five vectors composed of *n* elements: each element of each vector represents the coefficient of the corresponding order term of the polynomial to be solved for the *i*-th projected point. Several different algorithms are available to solve the 4th order polynomial, given its five coefficients. In this work, the general explicit formula is used [17] and all the needed operations are implemented in an element-by-element fashion on the five components of the coefficient vectors. The four solutions corresponding to the projected points are then obtained, all at once, as a 4 × *n* matrix. Again, the parameters *λ* can be obtained with element-by-element operations, and the proper solutions can be selected from the 4 × *n* matrix by exploiting the Matlab indexing functionalities. The solutions represent the values of the parameter *t* of the sine/cosine parametrization for each projected point. For this reason, the 3 × *n* matrix **X_r_**, containing the coordinates of the reflection points in the local reference systems, can be obtained through element-by-element calculation. Finally, **X_s_** is obtained by transforming **X_r_** through **R_g_^T^** and **T** (**X_s_** = **R_g_^T^X_r_** + **T**). The last re-projection step from the 3D reflection point to the corresponding pixel coordinates is performed by exploiting the functions available in the Matlab Camera Calibration Toolbox [18], which have been developed to handle 3 × *n* matrices of points.

**Figure 4:**
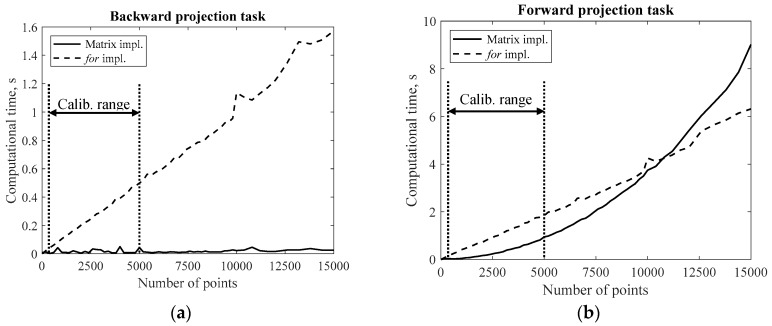
Code performances for an increasing number of points: (**a**) BP task and (**b**) FP task.

Two different implementations of the developed model could then be considered: a so-called *for*-loop implementation and a so-called matrix implementation. The *for*-loop implementation is obtained by repeating *n* times BP or FP functions applied to a single point (being *n* the number of points to be projected). The matrix implementation is obtained by running BP and FP scripts just once, giving as input a *k* × *n* matrix containing all the investigated points (*k* = 2 for BP and *k* = 3 for FP, independently from *n*). Generally, the comparison between different software implementations is achieved by means of Big-O analysis from an asymptotic point of view, i.e., by considering a number of projected points which increases till infinity. This analysis showed that the BP function is O(*n*) for both *for*-loop and matrix implementation. On the other hand, the FP function was found to be O(*n*) if the *for*-loop implementation is adopted, and O(*n*^2^) if the matrix implementation is adopted. This means that the *for*-loop implementation of the FP function outperforms the matrix implementation for a sufficiently high number of projected points *n*. Anyway, calibration procedures of catadioptric sensors are generally based on a number of points in the range 10^2^–10^3^. In this range, the asymptotic solution may be not fully representative, thus also a computational time comparison was performed. The performance of the proposed matrix implementation was tested by processing an increasing number of points from 1 to 15,000. The points were firstly processed altogether by exploiting the matrix format. Secondly, the considered points were processed singularly through a *for*-loop. This procedure was repeated for both BP and FP algorithms. The computation was performed on a workstation having a 64-bit operating system and 18 GB of RAM. The needed computational time was plotted against the number of projected points and reported in Figure 4: Figure 4a is referred to the BP function, while Figure 4b is referred to FP function. In both figures, the dashed line is referred to the *for*-loop implementation, while the solid line is referred to the matrix implementation.

The figure clearly shows that for both BP and FP tasks the *for*-loop implementation requires a computational time which linearly increases with the number of processed points (being the functions O(*n*)). The matrix implementation of the BP function also has a linear trend with respect to *n*, showing a slope that is smaller than the slope of the *for*-loop implementation, thus representing a time advantage for any number of processed points. On the other hand, the matrix implementation requires a computational time which increases more than linearly for the FP task, confirming the results of the Big-O analysis. Figure 4b shows that for a number of points lower than about 10^4^ (which is the common case for calibration purposes) the matrix implementation is faster than the *for*-loop implementation. Conversely, the *for*-loop implementation results faster than the matrix implementation for a number of points greater than 10^4^.

### 4.2. Jacobian Computation

The proposed analytical model has been developed for catadioptric vision systems. Several calibration strategies are available in literature [5,6,19,20], and all of them rely on a nonlinear optimization step to fine-tune the model parameters. In the field of nonlinear least squares optimization, a great advantage in terms of computational time is represented by the knowledge of the Jacobian matrix of the residual function to be minimized. Thus, in the present work, a great coding effort was spent to compute the analytical Jacobian of the developed functions, which contains the derivative values for each function output with respect to each function input. The present section is not aimed at providing a complete and detailed description of each derivative function. However, some hints about the Jacobian computation are given. The main difficulty is represented by the fact that the matrix-format solution requires the computation of the derivative of a matrix-function by a matrix-variable. In this case, the solution is found by deriving each component of the matrix-function (*m* × *n*) by each component of the matrix-variable (*p* × *q*). Each derivative can then be arranged in a large (and usually sparse) *mn* × *pq* matrix. In the present work, both matrix-function and matrix-variable are rearranged in a vectorial format as follows:

(10) $$\begin{array}{l} F_{M}=\left[\begin{array}{cccc} \left[\begin{array}{c} F_{1,1}\\ F_{1,2}\\ \vdots \\ F_{1,m} \end{array}\right] & \left[\begin{array}{c} F_{2,1}\\ F_{2,2}\\ \vdots \\ F_{2,m} \end{array}\right] & \cdots & \left[\begin{array}{c} F_{n,1}\\ F_{n,2}\\ \vdots \\ F_{n,m} \end{array}\right] \end{array}\right],\\ F_{V}={\left[\begin{array}{cccc} \left[\begin{array}{cccc} F_{1,1} & F_{1,2} & \cdots & F_{1,m} \end{array}\right] & \left[\begin{array}{cccc} F_{2,1} & F_{2,2} & \cdots & F_{2,m} \end{array}\right] & \cdots & \left[\begin{array}{cccc} F_{n,1} & F_{n,2} & \cdots & F_{n,m} \end{array}\right] \end{array}\right]}^{T}, \end{array}$$

(11) $$\begin{array}{l} V_{M}=\left[\begin{array}{cccc} \left[\begin{array}{c} V_{1,1}\\ V_{1,2}\\ \vdots \\ V_{1,p} \end{array}\right] & \left[\begin{array}{c} V_{2,1}\\ V_{2,2}\\ \vdots \\ V_{2,p} \end{array}\right] & \cdots & \left[\begin{array}{c} V_{q,1}\\ V_{q,2}\\ \vdots \\ V_{q,p} \end{array}\right] \end{array}\right],\\ V_{V}={\left[\begin{array}{cccc} \left[\begin{array}{cccc} V_{1,1} & V_{1,2} & \cdots & V_{1,p} \end{array}\right] & \left[\begin{array}{cccc} V_{2,1} & V_{2,2} & \cdots & V_{2,p} \end{array}\right] & \cdots & \left[\begin{array}{cccc} V_{q,1} & V_{q,2} & \cdots & V_{q,p} \end{array}\right] \end{array}\right]}^{T}, \end{array}$$

where the subscript “**M**” denotes the matrix-format and the subscript “V” denotes the vectorial format. This rearrangement can be easily obtained by using the Matlab function “*reshape*”. Each row of the matrix-derivative can then be obtained by deriving each element of *F*_V_ by each element of *V*_V_.

## 5. Validation

The proposed forward/backward projection models and their matrix implementation have been validated through a numerical analysis. Each function physically represents the inverse of the other, even if they were independently developed following two different analytical procedures. A check of the proposed procedure and its implementation can then be obtained by computing, for example, the backward projection of a group of points and verifying if their forward projection corresponds to the starting locations. Thus, a series of 2D reference points was processed through the backward projection function to determine the reflection directions. Then, several points were selected at a fixed distance on the reflection lines and re-projected through the forward projection function, thus obtaining a series of 2D coordinates. These coordinates were finally compared with the reference ones, in order to verify if the physical relation between the two functions was respected. A grid of 1,228,800 points was selected in the image plane, corresponding to each pixel of a camera having a 1280 × 960 resolution, as described below. The corresponding reflection directions were determined by the *cam2world* function. Then, a point at a distance of 400 mm was selected on each reflection direction, and its re-projection on the image plane was determined by the *world2cam* function. The distances between the re-projected points and the reference ones were computed, and a mean value of 3 × 10^−12^ pixels was found, which was considered to be mainly ascribed to numerical errors.

## 6. Application to Catadioptric Camera Calibration

**Figure 5:**
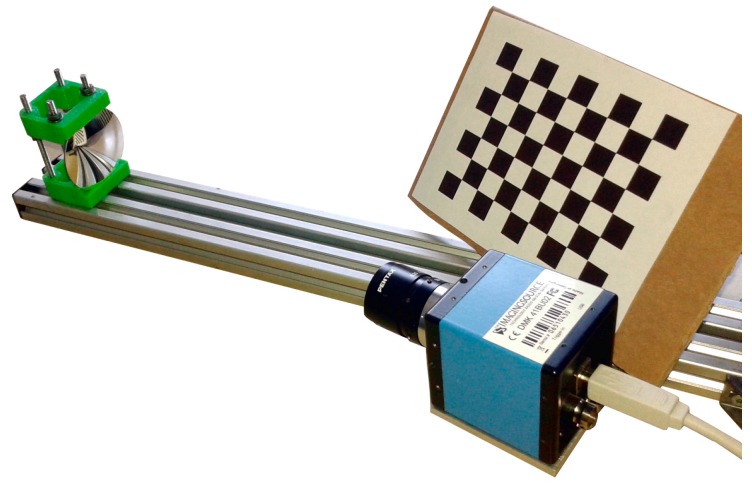
Catadioptric system with the planar chessboard pattern used for the experimental tests.

The proposed model has been used to calibrate a non-central catadioptric system composed of a perspective camera and a spherical mirror (Figure 5). The camera is a 8-bit monochrome charge coupled device (CCD) digital camera (The Imaging Source^®^, Taipei, Taiwan, DMK 41BU02, resolution 1280 × 960 pixels) equipped with a 16 mm focal length lens. The spherical mirror is provided with an enhanced aluminium coating (Edmund Optics, Barrington, NJ, USA, radius 50 ± 0.1 mm, solid angle 60°). The calibration procedure consists in the determination of the sphere’s radius and center with respect to the camera reference frame. The adopted calibration procedure firstly requires a calibration step to obtain the camera intrinsic parameters (radial and tangential distortions), which is performed by using the classical pinhole model [18]. The sphere’s calibration process is performed by acquiring a planar chessboard (having a 8 × 6 grid with a 12 mm square size) reflected in the mirror from several different positions and orientations (Figure 5).

The chessboard corners on the camera image plane were determined by exploiting the automatic algorithm described in [21]. The re-projection of the 3D chessboard corners on the camera image plane is univocally determined by the proposed FP model, given the center and radius of the spherical mirror. An optimization process can then be defined, with the optimization parameters represented by the actual sphere center and radius (four parameters) and the 3D coordinates of the chessboard corners. The target function to be minimized is represented by the sum of the quadratic distances between the re-projected corners and the detected ones for all the acquired chessboard placements. A theoretical virtual chessboard was defined (having a planar 8 × 6 grid with a square size of 12 mm) in the camera reference frame, having the first corner in the origin and laying on the *x*-*y* plane. The actual 3D coordinates of the chessboard can then be expressed by a rigid roto-translation of this theoretical grid, so that six parameters are needed for each acquired grid (three parameters to define the translation and three parameters to define the rotation). In this work, 15 images were acquired, so that 4 + 6 × 15 = 94 parameters were to be determined. The first guess for *c*_s_ was obtained by roughly measuring with a ruler the distance between the mirror and the camera, while the mirror nominal radius was used as a first guess for the parameter *r*. The initial guess for the poses of the chessboard patterns was unpractical to be measured (mainly due to the rotation). Thus, the grids were initialized to lay on the camera image plane with the first corner in the origin. A non-linear least square optimization procedure was used, exploiting the Trust-Region-Reflective algorithm, which allows to set upper and lower bounds for the parameters and to provide a Jacobian matrix to speed up computation. It is worth noting that the same algorithm, applied to the same optimization problem with or without an explicit evaluation of the Jacobian, requires a different computation time. The optimization algorithm needs to numerically estimate the Jacobian matrix when it is not explicitly provided, thus resulting in a time-consuming procedure. The Jacobian computation is useful only for optimization and calibration purposes, thus it is applied to a number of points which is typical of catadioptric systems calibration, i.e., 10^2^–10^3^. In this scenario, the asymptotic (Big-O) analysis may be not fully representative, thus the computation time comparison was performed to assess the code performances. Table 1 reports some comparison terms of the optimization process with or without the use of the Jacobian matrix. The relevant time difference between the two methods justifies the effort spent in the analytical Jacobian matrix determination. Moreover, the final optimization results are practically the same for the two procedures, thus validating the performed Jacobian analytical calculation.

**Figure 6:**
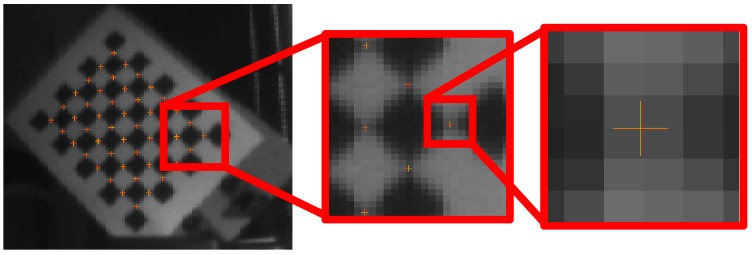
Example of grid re-projection on the image after the optimization process.

Figure 6 shows, as example, the results obtained on a single image. Yellow-crosses represent the chessboard corners as obtained by the automatic image processing algorithm, while red-crosses are the re-projected points as obtained by the proposed FP function.

**Figure 7:**
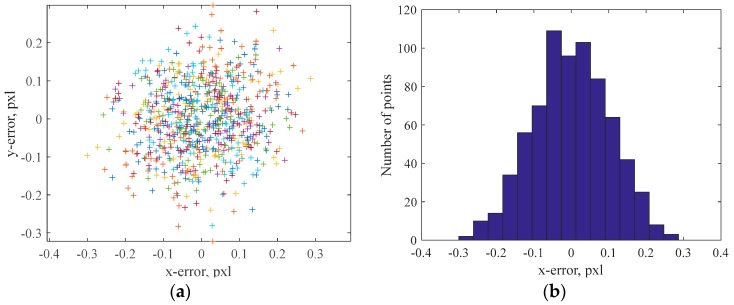
Re-projection errors after the optimization process: (**a**) 2D overview and (**b**) x-error histogram.

**Figure 8:**
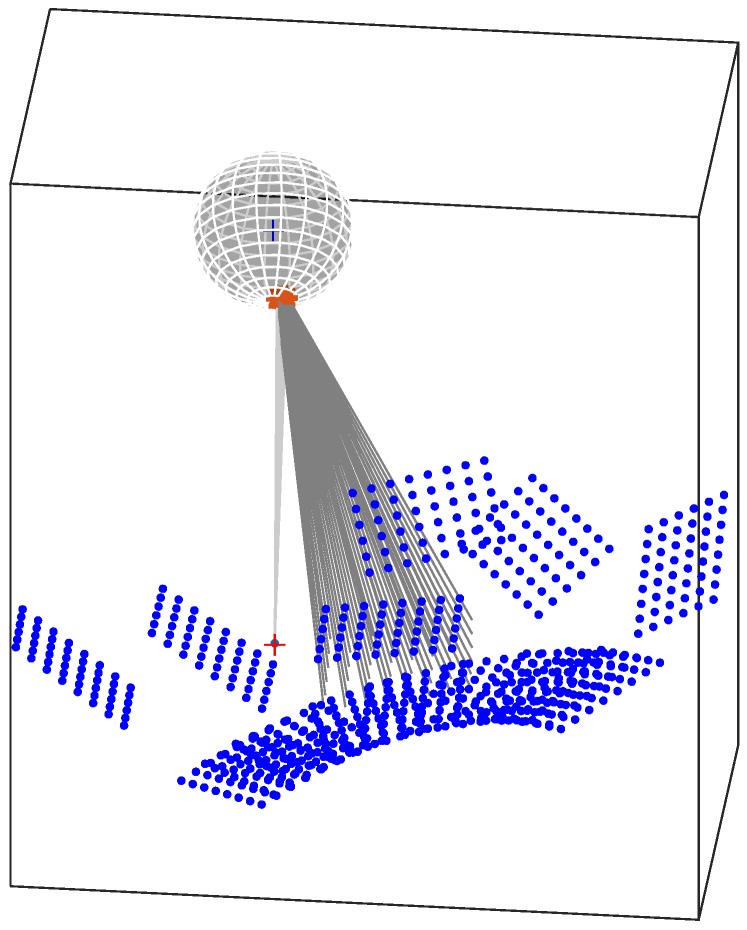
Optimized poses of the acquired grids.

The optimization results are also reported in Figure 7, which shows the distances between detected and re-projected grid corners for all the acquired images (Figure 7a) and the x-errors histogram (Figure 7b). The obtained maximum distance is about 0.32 pixel, while the mean distance (computed on 720 points) is 0.13 pixel. Figure 7 shows a Gaussian distribution of the error. This hypothesis has been verified through the Royton’s test [22], providing a *p*-value of 0.769 (with a significance of 0.05), which confirmed a Gaussian distribution of the experimental data. Finally, Figure 8 shows the poses of the acquired grids as obtained by the optimization procedure. The red-cross in the figure represents the camera central point, the sphere represents the mirror (with the proper scaling of positioning and radius) while the blue dots represent the grid-corners. The reflection rays for one of the grids were also represented, with a grey line, to show an example of the BP process.

Finally, a comparison between the proposed model and the model described in [6] was performed. The same image set was processed by using the toolbox associated to [6] and the reprojection errors were compared. A mean distance of 0.29 pixel was found, which is more than double with respect to the mean distance obtained with the proposed model. It is worth noting that the toolbox described in [6] relies on a SVP model, which is adapted for spherical mirror computation.

## 7. Conclusions

The present work describes the integration of a spherical mirror and a perspective camera in order to obtain a non-central catadioptric sensor for omnidirectional vision. A projection function between 3D points and corresponding 2D image points must be determined since the viewed scene undergoes a transformation due to the reflection in the mirror. This paper presents an analytical closed-form solution for both forward projection (FP) and backward projection (BP) tasks. An elegant formulation of the mathematical model for the FP solution is presented, along with a different parametrization of the equations with respect to approaches existing in literature. The analytical approach is firstly presented for the solution of a single-point reflection. Then, the implementation of the method in a matrix format is presented, in order to handle the computation in the case of point-clouds processing. Moreover, the Jacobian computation of both FP and BP functions was presented and briefly described since useful to speed up optimization processes typical of calibration tasks of imaging devices. Finally, the proposed functions have been applied to the calibration of a camera-mirror system, and exact coordinates of the mirror in the camera reference frame were determined, along with the sphere radius. The optimization process allowed to obtain the 3D pose of the acquired chessboard grids, which gave a Gaussian re-projection error and a mean re-projection distance of 0.13 pixels. The developed projection functions have been also implemented as Matlab scripts (“*cam2world.m*” and “*world2cam.m*”), which are available as supplementary material at [15].

## Figures and Tables

**Table 1:** Comparison of the optimization process performances with or without the Jacobian matrix.

	With Jacobian	Without Jacobian
Time	27 s	2301 s
Final target function	14.3 pxl^2^	
Max rep. dist.	0.3235 pxl	
Min rep. dist.	0.0099 pxl	
*c*_s_	0.1265 pxl	
*r*	[−1.9, −8.6, 284.3] mm	

**Figure A1:**
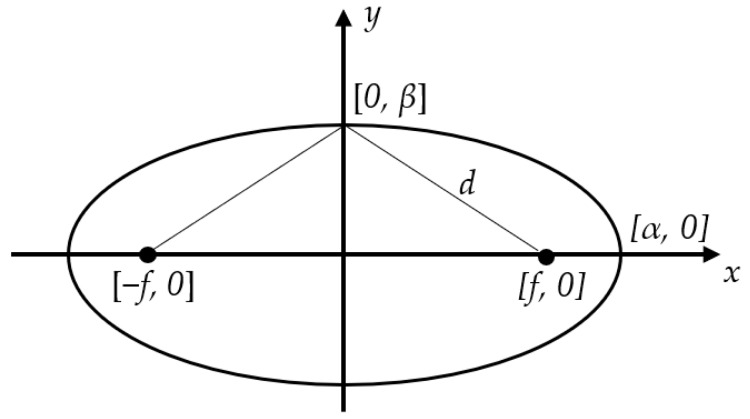
Ellipse parametrization.

## Appendix A

This Appendix shows the mathematical derivation of Equation (4), which is inspired to the solution described in [23]. The more generic equation to define a family of ellipses (and hyperbola) having the origin as midpoint between foci is:

(A1) $$\frac{x^{2}}{\alpha^{2}}+\frac{y^{2}}{\beta^{2}}=1$$

where *α* and *β* represent the intersection between the ellipse and the *x* and *y* axes respectively, as shown in Figure A1.

The sum of the distances between a point and the two foci must be equal for each point belonging to the ellipse, by definition. Thus, if the points [0, *β*] and [*α*, 0] are considered, it can be stated that:

(A2) $$2\sqrt{\beta^{2}+f^{2}}=2\alpha$$

Considering that in the chosen reference frame, and with the chosen scaling, the foci of the ellipse are the points [−1, 0] and [1, 0] (i.e., *f* = 1), Equation (A2) gives *α*^2^ *− β*^2^ = 1. It is then possible to parametrize Equation (A1) using the variable *λ* = *β*^2^ to obtain:

(A3) $$\frac{x^{2}}{\left(1+\lambda \right)}+\frac{y^{2}}{\lambda }=1$$

Which corresponds to the ellipse parametrization used in Section 3.2. Equation (A3) can be differentiated with respect to *x*, leading to:

(A4) $$\frac{2x}{\left(1+\lambda \right)}+\frac{2y}{\lambda }\frac{dy}{dx}=0\Rightarrow \frac{dy}{dx}=-\frac{\lambda }{\left(1+\lambda \right)}\frac{x}{y}$$

Equation (A4) represents the slope of the line tangent to the ellipse in the generic location [*x*, *y*]. The slope of the normal line is the negative reciprocal of the slope of the tangent, i.e., *y*(1 + *λ*)/(*xλ*). Thus, for each point [*x*, *y*] of the ellipse, the normal to the ellipse has the direction [*xλ*, *y*(1 + *λ)*]. The normal line passing by a generic point of the ellipse can be parametrized by the parameter *l* as:

(A5) {x(1+lλ),y(1+l(1+λ)):l∈ℝ}

In the case of an ellipse tangent to a circle, the normal to the ellipse in correspondence of the tangent point passes through the circle center [*a*, *b*] (in the current notation). Thus, from Equation (A5) it is possible to obtain:

(A6) $$\begin{array}{l} x\left(1+l\lambda \right)=a\Rightarrow l=\frac{a/x-1}{\lambda },\\ y\left(1+l(1+\lambda )\right)=b\Rightarrow l=\frac{b/y-1}{1+\lambda }. \end{array}$$

Combining the first and the second of Equations (A6) it is possible to state that:

(A7) $$\frac{a/x-1}{\lambda }=\frac{b/y-1}{1+\lambda }$$

Thus, the parameter *λ* can be deduced as *λ* = (*x* − *a*)*y*/(*ay* − *bx*), as reported in Section 3.2. Finally, this expression of *λ* can be substituted in Equation (A3) leading to Equation (4) in Section 3.2:

(A8) (ay−bx)(x(x−a)+y(y−b))−(x−a)(y−b)=0
